# Iridotomy, and Its Applicability as a Remedy for Certain Defects of the Eye, with Four Illustrative Cases

**Published:** 1875-06

**Authors:** A. W. Calhoun

**Affiliations:** Professor of Diseases of the Eye and Ear in the Atlanta Medical College


VOL. V.—JUNE, 1875.—NO. 6.

### Original Communications.

### IRIDOTOMY, AND ITS APPLICABILITY AS A REMEDY FOR CERTAIN DEFECTS OF THE EYE, WITH FOUR ILLUSTRATIVE CASES.

BY A. W. CALHOUN, M.D., *Professor of Diseases of the Eye and Ear in the Atlanta Medical College.*

*Read before the Medical Association of Georgia, in Savannah, April 21st,* 1875.

The operation bearing the above name has long been known, and even to some extent, though in a rude way, put into practice; but to DeWecker, of Paris, belongs principally the honor of having perfected and brought prominently before the profession the operation as it now exists, and of having so applied it, that it recommends itself under certain circumstances as a substitute for the old operation of iridectomy.

An iridectomy is the *cutting out* a greater or less portion of the iris; an iridotomy is the *cutting into* the iris, but removing no portion, yet without the loss of any of the tissue, accomplishing the same purpose, viz.: the entrance of light into the interior of the eye through an artificial pupil. It needs no argument to demonstrate that of two modes of operating, that is best which attains the same good end with the least sacrifice of the parts operated upon. If *incising* a diseased finger answered the same and perhaps a better purpose than the *excision* of the member, but few moments would be consumed in deciding upon the proper mode of procedure.

If an *incision* into the iris does a service equal to an *excision* of a portion of it, there should be no hesitation in resorting to the first, even though it should possibly require the more skill and care of the two. Ocular defects demanding the formation of an artificial pupil have heretofore been always met by an iridectomy, but the recent advances in ophthalmic surgery teach that this was in many instances a useless expenditure of tissue, and that an iridotomy would have done the same, whereby many inconveniences and disadvantages attending the first could have been easily avoided.

In making an iridectomy, it is often desirous to have the opening in the iris as small as possible, for when the operation is made with the view simply of letting the rays of light pass through into the interior of the eye, a large iridectomy, to a great extent, defeats the very object it is desired to accomplish, by permitting the passage of too much light, and thereby producing, so to speak, a confusion of vision. An iridotomy meets this objection thoroughly, as simply a slit is made into the iris, and only so great an opening is produced as is brought about by the contractility of the parts, separating the cut edges of the iris, usually to a slight but sufficient degree.

The four cases I have recently operated upon, and which I now report, demonstrate fully the advantages of the more recent operation (the iridotomy) over that usually made (the iridectomy) for the same troubles.

Case I.—C. C., of Florida, æt. 58, and in good health, was operated upon in November, 1874, by Graefe's Linear Extraction, for the removal of cataract from the left eye. From some cause, previous to the operation, the lens had become partially dislocated and immediately upon finishing the incision through the upper corneo-sclerotic junction, and upon the outflowing of the aqueous humour, the displaced lens presented itself at the edge of the wound and was at once removed, even before the iridectomy, which is a part of this operation, could be made.

Instead of the iris prolapsing after the exit of the lens, it turned backwards into the vitreous humour towards the ciliary bodies. As the vitreous was soft and threatened to escape in a dangerous quantity, it was thought best not to trouble the iris with the view of cutting out a portion of it, particularly since it was well out of the wound, and left the pupil in tolerable shape and position. The wound healed nicely, and by the use of atropine, the iris with the pupil, was brought back into proper place. Several days later, by an unlucky movement and a violent strain on the part of the patient, the corneo-sclerotic wound was ruptured, and a prolapsus of all that part of the iris took place, corresponding to the inner edge of the wound. Severe inflammation followed, and upon recovery it was found that the large prolapsus was adherent to the edges of the wound, and the pupil drawn so high up towards the upper corneal border and underneath the upper lid, that the patient could only make out objects at *any* distance very indistinctly. Chloroform was given, the lids held apart by an assistant and the prolapsed iris clipped away close up to the cornea, and an iridotomy made in the following manner:

Through the opening made by cutting away the prolapsus, the closed blades of DeWecker's scissors were passed into the anterior chamber as far as the lower pupillary border; here the blades were allowed to separate, and one passed through the pupil and behind the iris, the other in front of the iris, and the instrument pushed gently downwards towards the bottom of the anterior chamber, until the larger portion of the iris, between its pupillary margin and its periphery, was caught between the blades, which upon being brought firmly together, made a straight incision through the substance of the iris, severing the circular muscular fibres (the sphincter), the divided ends of which contracting, drew the two cut surfaces away from each other, leaving a triangular opening with the base upwards toward the pupil, and the apex downwards toward the periphery of the iris or bottom of the anterior chamber.

Under the ordinary treatment, the corneo-sclerotic wound healed rapidly, the iridotomy permitting the free entrance of light into the eye. Upon being dismissed a little later, his vision was $\frac{2}{3}$ with No. 4 double convex (or cataract) glass, and with No. $2\frac{1}{2}$ cataract glass he read small type.

Case II.—Wm B., of Georgia, æt. 25. This patient has had gran ophthalmia in both eyes, producing a deep ulcer upon the centre of the left cornea. By persistent treatment with nitrate silver, sulph. copper, etc., the granulations have disappeared, and the corneal ulcer in the left eye has healed; but there exists in its stead a thick opacity, situated directly in front of the pupil, and interfering materially with good vision. Dilating the pupil and examining the fundus of the eye with the ophthalmoscope through the clear portion of cornea, everything within was found to be perfectly normal, and with the pupil in this dilated condition, the vision (particularly for distant objects) was immensely improved, the rays of light passing without hindrance through the transparent parts of the cornea. 'Twas evident that an artificial pupil would greatly increase the vision, and with this in view, the iridotomy was decided upon as soon as the pupil had contracted to its original size, early in December, 1874. The operation in this case required still greater care and caution than the other, on account of the presence of the sound lens, which, as will be remembered, had become cataractous, and been extracted in the first case, before the necessity for the iridotomy arose. With the lids held apart and the eye securely fixed, an opening was made through the upper corneo-sclerotic junction, by means of a spear-shaped iridectomy knife, and through this opening the closed blades of De Wecker's scissors were advanced into the anterior chamber till the lower margin of the pupil was reached. Opening the blades, one was passed through the pupil behind the iris and resting upon the anterior capsule of the lens, the other in front of the iris, and the instrument forwarded until there rested between the blades a sufficient amount of iris, which was divided, and at once a free triangular opening was made by the contractility of the parts separating the cut edges.

When seen on the following day, and after the anterior chamber had refilled, the cut edges of the iris had drawn still farther apart, and a sufficiently large artificial pupil was formed below the corneal opacity to permit of excellent sight. There was no accurate test of vision made, yet the patient, as well as myself, was daily convinced of its great improvement. I have recently heard from him, and to the effect that his vision has been steadily improving.

Case III.—J. L. G., of N. C., æt. 27. In February 1875, an iridotomy was made upon this patient for relief from exactly the same trouble as in the last. He had had gran ophthalmia for several years, and upon the left cornea an ulcer had formed. After curing the granulations and the ulcer, there remained behind, a central corneal opacity, which, as is often the case, had rendered him myopic (near sighted), and even for near objects vision was indistinct, and there was an "*overstrained*" feeling about the eye, whenever he attempted to fix his vision upon anything. The utmost extent of his vision was $\frac{2}{7}$, and this was rather uncertain.

The lens being also present here, the same precautions were necessary, and precisely the same steps were gone through with in making the operation, as in the previous case. No undue inflammation followed the operation, and upon a re-accumulation of the aqueous humour, a large opening existed in the iris below the opacity. The patient was dismissed the latter part of March with vision amounting to $\frac{2}{5}$, perfectly clear and distinct. But more particularly for distance was the improvement most marked, small objects being readily recognized now, which before the operation were not seen at all, or only their outlines made out. The "*overstrained*" feeling (as the patient described it) was totally removed, no doubt by the free, unobstructed entrance of light into the eye.

Corneal opacities not unfrequently are the causes of a greater or less degree of myopia, and this case is particularly interesting, on account of this defect of refraction being to a great extent relieved by the operation.

CASE IV.—Miss C. of Ga., æt. 35. In this lady mature cataract had made its appearance at an unusually early age. The lens was removed from the right eye by means of Graefe's linear extraction, in which a large upward iridectomy was made. On the 3d or 4th day after the operation, a slow subacute iritis set in, lasting for several weeks and resulting in the complete occlusion of the natural and artificial pupils by the formation of a thick exudative membrane, producing what is ordinarily known as secondary cataract, and the drawing of the pupil high up underneath the upper lid. Vision, which was good immediately after the operation, was now gone.

Several months after the extraction, the iridotomy was performed. An opening was made through the upper part of the cornea, with the spear-shape knife, the point of which, on entering the anterior chamber, was also made to puncture the membrane filling the pupil. One blade of the iridotomy scissors was passed through the puncture behind the membrane, the other in front, and pressed downward till the whole width of the membrane and a large part of the iris were brought between the two blades, which being closed, divided both the obstructing membrane and the iris. As the two sides of the iris drew away from each other, they carried with them the corresponding sides of the divided membrane, forming a large artificial pupil through which the light could pass unobstructed. The patient could immediately count fingers without hesitation, distinguishing between the different ones with accuracy.

While waiting for the eye to recover thoroughly from the effects of the operation, before making a scientific test of the vision by means of the test types, she was called suddenly home, and the opportunity for getting very exactly the degree of vision, was lost; still it is sufficient to say that before leaving, with No. 4 cataract glass, she had been walking about without assistance, seeing as well as one usually does at this time; after the successful removal of cataract. With No. 2½ cataract glass she was able to read print, a size larger than the ordinary print of newspapers.

After a completely successful extraction of cataract, the vision is constantly improving for several months. Upon the iridotomy proving successful in this instance, it was simply successfully completing the cataract operation, and I have no doubt, indeed have been informed, of the gradual increase in vision for several months after the operation.

In each of the foregoing cases it will be seen, that where the operation (the iridectomy) usual for the relief of such troubles was indicated, the *iridotomy* was made instead, and with gratifying success in every instance.

To repeat then, this operation recommends itself from the fact that no part of the iris tissue is destroyed or taken away, and that the artificial pupil thus made, is sufficiently small to prevent the letting in of too much light. This latter is, in particular, a great point gained. There is usually no difficulty in making a large pupil, but on the contrary, to get it small enough to concentrate the rays of light, in some degree, and thereby overcome the confusion produced by the rays scattering after passing through a large opening, is oftentimes by no means an easy matter.

It is somewhat difficult to perform it, in those cases where the lens is present, for one blade of the instrument (the scissors) must necessarily rest upon the anterior capsule of the lens, finding its way slowly down between this and the posterior surface of the iris, for when the corneal or corneo-sclerotic opening is made, the aqueous humor rushes out, and the lens and the iris on its posterior surface, are forced together. The danger is in wounding the lens or its capsule and producing traumatic cataract, but with the requisite care and skill, there should be but little fear of this taking place. Where the lens has already been removed, we have no such trouble to contend with, and the operation is much more easily performed.—One other precaution is specially necessary, viz.: to replace the prolapsed portion of iris which juts out of the external wound as the aqueous humor flows out. This is readily replaced by using a blunt pointed stylet.

In cases of lamellar cataract, where the margin of the cortical substance is perfectly clear (which can be more distinctly seen when the pupil is widely dilated), the iridotomy would answer an admirable purpose. The lens is not sufficiently opaque to be extracted, or to cause its absorption by means of discission or laceration; but the only thing necessary to give clear vision is to make a small cut (a slit) through the iris so as to allow the light to impinge upon the transparent portion of the lens. The iridotomy would do this perfectly, and avoid the *confusion of sight* which would most probably be produced, should a part of the iris be clipped off.

*Central capsular cataract* is occasionally so large as to almost entirely fill the pupil when of normal size. It is necessary to do something in order to allow the child the proper exercise of the eye. Here, also, the iridotomy would certainly be preferred to the usual modes of operating.

The operation is particularly serviceable in those cases where iritis or irido-cyclitis has followed cataract operations by extraction, and as a consequence, a more or less thick membrane (secondary cataract) has filled up the natural pupil and the iridectomy. These membranes are often so tough and firm that it is dangerous to exercise sufficient power with the needle to break it up, or even to make a single rent through it. All who have had such cases to treat, can well recall to mind how often they have been compelled to make operation after operation, before setting aside the obstacle.

The iridotomy leaves a single cut through both the membrane and the iris, (the pupil being drawn upwards by the contraction of its tissue and the membrane), and the sphincter muscle acting, separates the cut edges of the two, one from the other, and forms an opening of greater or less size. This avoids a second iridectomy, which is frequently made under such circumstances.

Diseases of the lids as well as of the cornea, not unfreqently result in permanent opacities upon the cornea, which obstruct the vision more or less, according to their situation, covering the pupil in part or entirely. In many of these cases, it is only necessary to change the position of the pupil to a point behind a transparent portion of the cornea, to dispel the cloud which may have so long shut out the bright light from the diseased eye, and permit of clear, almost perfect vision. For the accomplishment of this, the iridectomy has hitherto been almost the sole remedy, notwithstanding that results often followed it which were extremely disturbing in their nature. The iridotomy affords the same good, and but few if any of the subsequent confusing results take place, but it requires greater caution and possibly more skill than the iridectomy.

There are cases, of course, where the latter operation cannot be dispensed with, but the iridotomy is by far preferable whenever a small artificial pupil is desired, or in those cases where to remove a portion of the iris would originate inconveniences almost if not quite counterbalancing the advantages.

